# Succinyl-κ-carrageenan Silver Nanotriangles Composite for Ammonium Localized Surface Plasmon Resonance Sensor

**DOI:** 10.3390/polym14020329

**Published:** 2022-01-14

**Authors:** Mohd Hafiz Abu Bakar, Nur Hidayah Azeman, Nadhratun Naiim Mobarak, Nur Afifah Ahmad Nazri, Tengku Hasnan Tengku Abdul Aziz, Ahmad Rifqi Md Zain, Norhana Arsad, Ahmad Ashrif A. Bakar

**Affiliations:** 1 Photonics Technology Laboratory, Department of Electrical, Electronic and Systems Engineering, Faculty of Engineering and Built Environment, Universiti Kebangsaan Malaysia, Bangi 43600, Selangor, Malaysia; hafiz.bujei95@gmail.com (M.H.A.B.); afifahnazri063@gmail.com (N.A.A.N.); noa@ukm.edu.my (N.A.); 2 Department of Chemical Sciences, Faculty of Science and Technology, Universiti Kebangsaan Malaysia, Bangi 43600, Selangor, Malaysia; nadhratunnaiim@ukm.edu.my; 3 Institute of Microengineering and Nanoelectronics, Universiti Kebangsaan Malaysia, Bangi 43600, Selangor, Malaysia; hasnanaziz@ukm.edu.my (T.H.T.A.A.); rifqi@ukm.edu.my (A.R.M.Z.); 4 Institut Islam Hadhari, Universiti Kebangsaan Malaysia, Bangi 43600, Selangor, Malaysia

**Keywords:** polysaccharide, carrageenan, surface plasmon resonance, ammonium ion, adsorbent, isotherm

## Abstract

This research investigates the physicochemical properties of biopolymer succinyl-κ-carrageenan as a potential sensing material for NH_4_^+^ Localized Surface Plasmon Resonance (LSPR) sensor. Succinyl-κ-carrageenan was synthesised by reacting κ-carrageenan with succinic anhydride. FESEM analysis shows succinyl-κ-carrageenan has an even and featureless topology compared to its pristine form. Succinyl-κ-carrageenan was composited with silver nanoparticles (AgNP) as LSPR sensing material. AFM analysis shows that AgNP-Succinyl-κ-carrageenan was rougher than AgNP-Succinyl-κ-carrageenan, indicating an increase in density of electronegative atom from oxygen compared to pristine κ-carrageenan. The sensitivity of AgNP-Succinyl-κ-carrageenan LSPR is higher than AgNP-κ-carrageenan LSPR. The reported LOD and LOQ of AgNP-Succinyl-κ-carrageenan LSPR are 0.5964 and 2.7192 ppm, respectively. Thus, AgNP-Succinyl-κ-carrageenan LSPR has a higher performance than AgNP-κ-carrageenan LSPR, broader detection range than the conventional method and high selectivity toward NH_4_^+^. Interaction mechanism studies show the adsorption of NH_4_^+^ on κ-carrageenan and succinyl-κ-carrageenan were through multilayer and chemisorption process that follows Freundlich and pseudo-second-order kinetic model.

## 1. Introduction

Ammonia (NH_3_) is an essential source of nitrogen for marine phytoplankton [1]. It is readily dissolved in water to produce ammonium ions (NH_4_^+^). An excessive amount of NH_4_^+^ in water leads to significant ecological effects such as algae bloom in the water system, oxygen reduction in water, and eventual ruin of the ecological balance of the water system. High levels of NH_4_^+^ in water systems come mainly from agricultural activities, industrial factories, mines, and sewage pollution [2].

The localized surface plasmon resonance (LSPR) sensing method is an evergreen technology and a susceptible optical method for chemical and biochemical analysis. LSPR uses collective free electron oscillation in metallic nanoparticles driven by an electromagnetic wave from incident light of a specific wavelength at the monometallic surface [3,4]. LSPR has been widely used for cationic detection such as Pb^2+^, Cr^6+^, and Fe^2+^. Incorporation of LSPR with a sensing material such as polymers for enhancement of LSPR performance is vital. Many researchers used polysaccharides due to their polycationic nature, which easily attaches metal nanoparticles to their surface via electrostatic interaction [4,5]. Because of their inexpensive, biodegradable, non-toxic, and environmentally friendly properties, carrageenan is the most appealing polymer among natural polymeric materials.

κ-carrageenan can be extracted from red seaweeds, where it is made up of a sulphate group per disaccharide unit with 4-linked 3,6-anhydro-α-D-galactose and 3-linked-β-D-galactose repeating units [6]. Recently, κ-carrageenan has been extensively used in research due to the high number of active sites such as hydroxyl and sulphate groups. κ-carrageenan has been studied for heavy metal adsorbent in LSPR sensors, mercury ion colorimetric detectors, and as a host in polymer electrolyte application [7]. Because of the existence of extra oxygen atoms provided by hydroxyl (O-H) and sulfate (O=S=O) groups in the κ-carrageenan structure, the sensor performance of LSPR is better when utilising κ-carrageenan as a sensing material in comparison to chitosan [3]. These groups enable the formation of a coordination bond with cation. To further improve the performance of κ-carrageenan as a sensing material, chemical modification is necessary to achieve this objective. The effect of hydroxyl group substitution was studied with other oxygen-rich substituents to coordinate with cation [8]. The functional group’s substitution in κ-carrageenan structure can enhance its physiochemical properties. When compared to pristine carrageenan, modified carrageenan’s ionic conductivity may increase by three orders of magnitude.

Bakar et al. successfully synthesized succinyl-κ-carrageenan by reacting succinic anhydride with κ-carrageenan in a one-step reaction method [7]. It was reported that succinyl-κ-carrageenan increased the performance of the polysaccharide. It facilitated the degree of ion dissociation and ion mobility since the electronegative atom’s addition increases the interaction with cation in a salt solution. The addition of the succinyl group in κ-carrageenan provides two carbonyl moieties where it gives more electronegative sites for interaction than the carboxymethyl group. Thus, it can be further studied as a potential active material for NH_4_^+^ interaction in the LSPR sensing technique.

Therefore, we report a novel, triangular AgNP LSPR sensor based on biopolymer succinyl-κ-carrageenan for the detection of NH_4_^+^ ions. Due to the increased electronegative active site in succinyl-κ-carrageenan, it is expected the succinyl-κ-carrageenan operates better than the pristine κ-carrageenan LSPR sensor. The succinyl-κ-carrageenan was established in a report by Bakar et al. [7]; thus, molecular characterization is not reported in this work. This research focuses on studying the physicochemical properties of succinyl-κ-carrageenan and evaluating its performance as a potential active material for the NH_4_^+^ LSPR sensor. The succinyl-κ-carrageenan’s properties were investigated and compared with pristine κ-carrageenan using Atomic Force Microscopy (AFM) and Field Emission Scanning Electron Microscope (FESEM). Then, the LSPR sensor performance utilizing the suggested sensing material to detect NH_4_^+^ was evaluated. Finally, the isotherm and kinetic study were conducted to analyse and predict the succinyl-κ-carrageenan surface interaction mechanism with NH_4_^+^.

## 2. Materials and Methods

### 2.1. Material

Industrial-grade κ-carrageenan was taken from Tacara Sdn Bhd (Sabah, Malaysia) (TA150) (Mw: 12 kDa). Succinic anhydride, (3-Aminopropyl) triethoxysilane (APTES), acetone, acetonitrile, silver nitrate (AgNO_3_), ammonium chloride, trisodium citrate, sodium borohydride (NaHB_4_), hydrogen peroxide (H_2_O_2_), calcium nitrate tetrahydrate, manganese sulfate monohydrate, zinc nitrate hexahydrate, and ammonium chloride (NH_4_Cl) were bought from Sigma–Aldrich. Lead standard solution and iron nitrate nonahydrate were bought from Merck. All solvents were used without purification.

### 2.2. Preparation of Succinyl Kappa-Carrageenan

Succinyl-κ-carrageenan was synthesised according to the previous technique established in [7]. One gram of κ-carrageenan was dissolved in 200 mL of distilled water and transferred into the conical flask. Two grams of succinic anhydride was dissolved in 20 mL of acetone and dropped into the flask at room temperature for 30 min. The reaction was continued at 40 °C for 24 h. The mixture was cooled down to room temperature once the reaction was completed. Additional acetone was added to the mixture before acetonitrile to precipitate the compound. The unreacted succinic anhydride can be eliminated by filtering and washing the precipitate with acetonitrile. Finally, the precipitate was dried in a desiccator for 12 h.

### 2.3. Preparation of AgNP

Preparation of AgNP followed the procedure established by [9,10]. An amount of 0.2 mL of the 0.05 M AgNO_3_ solution was added into deionized water (96.56 mL). The mixture was stirred at 900 rpm. Then, 2.0 mL of 75 mM trisodium citrate and 0.24 mL of 30% H_2_O_2_ were added into the mixture. Next, 1.0 mL of NaBH_4_ was immediately injected into the mix. The colour of the mixture was observed until the dark blue colour of the AgNP solution formed. Lastly, the dark blue solution was centrifuged at 4000 rpm for 1 h to obtain the dark blue sediment and collected to acquire about 2 mL of the blue sediment.

### 2.4. Preparation of AgNP LSPR Glass Slide via the Composite Method

The substrate was prepared using 1 cm × 1 cm quartz glass. The substrate was washed using liquid detergent and deionised water. Then, the substrate was immersed in piranha solution (1:3 = H_2_O_2_:H_2_SO_4_) for an hour. Later, the substrate was immersed and sonicated in acetone and ethanol for 15 min, respectively. Next, the substrate was immersed in 5% APTES solution for another hour. Rinse the substrate with ethanol and dry it on a hotplate at 100 °C for 30 min.

**Figure 1:**
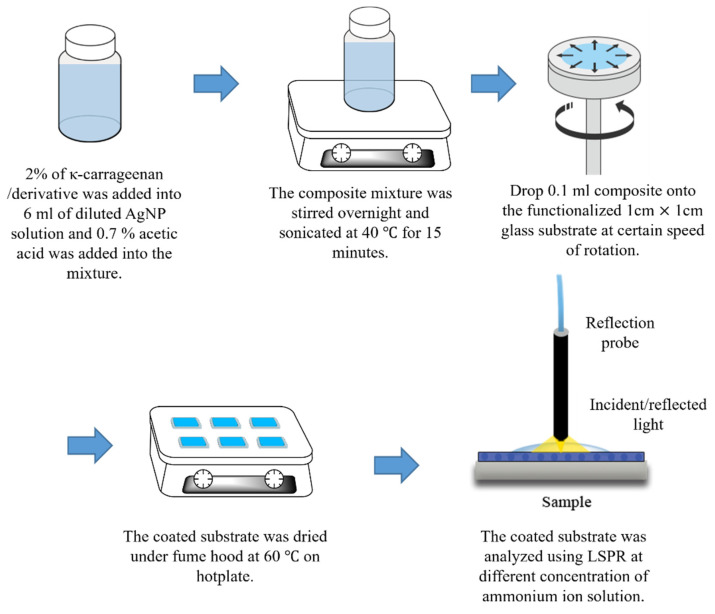
Experimental procedure for preparation of substrate coated with AgNP-κ-carrageenan and AgNP-succinyl-κ-carrageenan composites.

Meanwhile, AgNP-succinyl-κ-carrageenan and AgNP-κ-carrageenan composites were prepared by mixing 2% of succinyl-κ-carrageenan and κ-carrageenan into 6 mL of diluted AgNP sediment. Then about 0.7% of glacial acetic acid was added to the mixture to help in dissolving the polymer. The mixture was stirred overnight and sonicated at 40 °C for 15 min to dissolve the compounds. Then, 0.1 mL of the composite was coated on a 1 cm × 1 cm glass slide using a spin coater with 1500 rpm and dried on a hot plate. The process was repeated twice. The coated substrates were analysed using LSPR. Figure 1 depicts the procedure preparation of the glass slide using the composite mixture.

### 2.5. Experimental Setup for Detection of NH_4_^+^ Ion Using LSPR Sensor

In this research, the method of measurement using reflectance LSPR was more suitable due to the nature of carrageenan and its derivative since pristine κ–carrageenan and succinyl-κ–carrageenan is non-transparent [3]. NH_4_^+^ could be easily detected by dropping the NH_4_^+^ solution on the surface of AgNP-κ–carrageenan and AgNP-succinyl-κ–carrageenan substrate for LSPR analysis.

**Figure 2:**
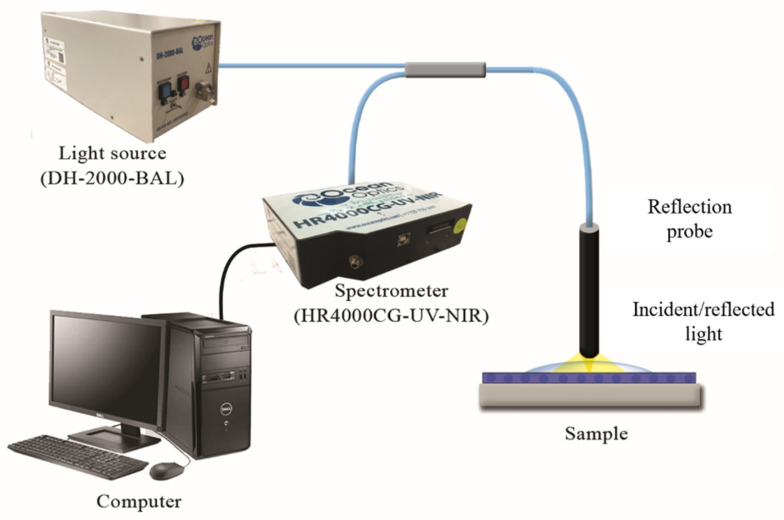
Experimental setup for detection of NH_4_^+^ using LSPR sensor.

The system consisted of a light source from Ocean Optic (DH-2000-BAL, Ocean Optics Inc., Dunedin, FL, USA) and a light sensor (HR4000CG-UV-NIR) from Ocean Optics (Dunedin, FL, USA to measure the optical response. The fibre core of the reflection probe used in this experiment was 600 μm with a numerical aperture of 0.22. A reflection probe was connected to the light source and spectrometer. The probe was adjusted to an optimum distance above the sample, as shown in Figure 2.

### 2.6. Characterisation

The morphology properties of succinyl-κ-carrageenan and κ-carrageenan were analysed using FESEM coupled D8 Quest SC-XRD (Bruker, MA, USA) at 15 Kv with a magnification of 5000×, 20,000×, and 50,000×. The surface roughness of succinyl-κ-carrageenan and κ-carrageenan was investigated using AFM NX-10 (Park Systems Corp., Suwon, Korea). The XE Image Processing software (Park Systems Corp., 4th Edition) was used to obtain average surface roughness. Measurements are based on the heights of peaks and valleys on the surface.

### 2.7. Interaction Mechanism Study

The procedure of NH_4_^+^ detection was conducted using the standard method Nesslerisation by UV-Visible absorbance. NH_4_Cl was utilised as an analyte. A 1000 ppm stock solution of NH_4_^+^ as made by diluting 1.486 g of dried NH_4_Cl salt in 500 mL of deionised water. Then, a series of dilutions ranging from 0.05 ppm to 10 ppm were prepared.

The effective dosage on the adsorption capacity of NH_4_^+^ were investigated in 20 mL of 1 ppm NH_4_^+^ solution for 60 min as standardised equilibrium time for both compounds by varying the mass of compounds at 360 rpm. Meanwhile, the initial NH_4_^+^ concentration effect was conducted by varying the concentration 20 mL of NH_4_^+^. NH_4_^+^ was investigated within the range of 0.05–2.0 ppm at 360 rpm for 60 min using 0.1 g as an optimum adsorption mass for κ-carrageenan and succinyl-κ-carrageenan. Meanwhile, the effect of interaction time between pristine κ–carrageenan and succinyl-κ-carrageenan on the adsorption capacity of NH_4_^+^ was investigated by varying the interaction time from 10–120 min.

The mixture was filtered using a 0.22 µm membrane syringe filter, rinsed, and the filtrate was transferred into a round-bottomed flask. Next, 1 mL of dechlorinating agent and 2 mL of borate buffer was added to the mixture. The mixed solution was then 75% distilled into 5 mL of 0.3 M sulfuric acid using Kjeldahl apparatus to remove any trace of another compound. A few drops of 0.05 M NaOH was added to the sample to neutralise the sample. An amount of 1.2 mL of Nessler Reagent was dropped into the collected sample to develop the yellow-orange solution. UV-Vis spectrometer (OceanOptic^®^) was used to measure the absorbance of the complex solution at 425 nm using 1 m × 1 m cuvette. The data from the absorbance experiment were collected and analysed to study the isotherm properties based on Langmuir and Freundlich model. The amount of adsorbed NH_4_^+^ onto carrageenan compound at equilibrium time, *q_e_* (mg/g), was calculated using the following equation:

(1) $$q_{e}=\frac{C_{0}-C_{e}}{m}\times V$$

where *C*_0_ (mg/L) is the initial concentration of NH_4_^+^, *C_e_* (mg/L) is the NH_4_^+^ concentration at equilibrium time, *V* (L) is the volume of NH_4_^+^ used, and *m* (g) is the mass of pristine κ–carrageenan and succinyl-κ-carrageenan.

The Langmuir isotherm assumes monolayer adsorption onto an adsorbent surface. The linear form of Langmuir adsorption isotherm can be expressed as [11,12]:

(2) $$\frac{c_{e}}{q_{e}}=\frac{1}{q_{max}K_{L}}+\frac{c_{e}}{q_{max}}$$

where;

*K_L_* = adsorption equilibrium constant (L/mg)
*q_max_* = maximum ammonium ion adsorption capacity (mg/g)
*q_e_* = adsorption capacity (mg/g) at equilibrium
The value of *q_max_* is obtained from a linear plot of *C_e_*/*q_e_* versus *C_e_*.

Meanwhile, the Freundlich isotherm model described adsorption on heterogeneous systems and assumed that the adsorption occurs on-site with different adsorption energies. The adsorption occurs in multilayer, and the Freundlich isotherm equation was given as follows [11,12]:

(3) $$q_{e}=K_{F}C_{e}^{\frac{1}{n}}$$

This equation could be written in linear form as follows:

(4) $$logq_{e}=logK_{F}+\frac{1}{n}logC_{e}$$

*K_F_* = adsorption capacity
*n* = adsorption intensity, respectively.
*K_F_* and *n* are obtained from the slope and intercept of the linear *log q_e_* versus log *C_e_*.

The effect of stirring time was investigated by using 0.1 g of κ-carrageenan and succinyl-κ-carrageenan as optimum adsorption mass of NH_4_^+^. The adsorption was investigated using 20 mL of 2.0 ppm as the optimum concentration of NH_4_^+^ at 25 °C at 360 rpm for desired stirring time. The amount of adsorbed NH_4_^+^ onto carrageenan compound at a time, *q_t_* (mg/g), was calculated using the following equation:

(5) $$q_{t}=\frac{C_{0}-C_{t}}{m}\times V$$

where *C*_0_ (mg/L) is the initial concentration of NH_4_^+^, *C_t_* (mg/L) is the NH_4_^+^ concentration at the time, *t* (min), *V* (L) is the volume of NH_4_^+^ used, and *m* (g) is the mass of pristine κ–carrageenan and succinyl-κ-carrageenan.

To examine the kinetic mechanism, the pseudo-first-order and the pseudo-second-order kinetic models were used for analyses and comparison. The pseudo-first-order rate equation are as follows [13,14]:

(6) $$log\left(q_{e}-q_{t}\right)=logq_{e}-\frac{k_{1}t}{2.303}$$

The pseudo-second-order rate equation was given as below [13,14]:

(7) $$\frac{t}{q_{t}}=\frac{1}{k_{2}q_{e}^{2}}+\frac{t}{q_{e}}$$

where;

*q_t_* = the adsorption capacity at time t (min).
*k*_1_ = adsorption rate constants of pseudo-first-order (min^−1^)
*k*_2_ = pseudo-second-order adsorption rates (g mg^−1^ min^−1^)

## 3. Results and Discussion

This section may be divided by subheadings. It should provide a concise and precise description of the experimental results, their interpretation, as well as the experimental conclusions that can be drawn.

### 3.1. Surface Studies of Sensing Materials for LSPR

**Figure 3:**
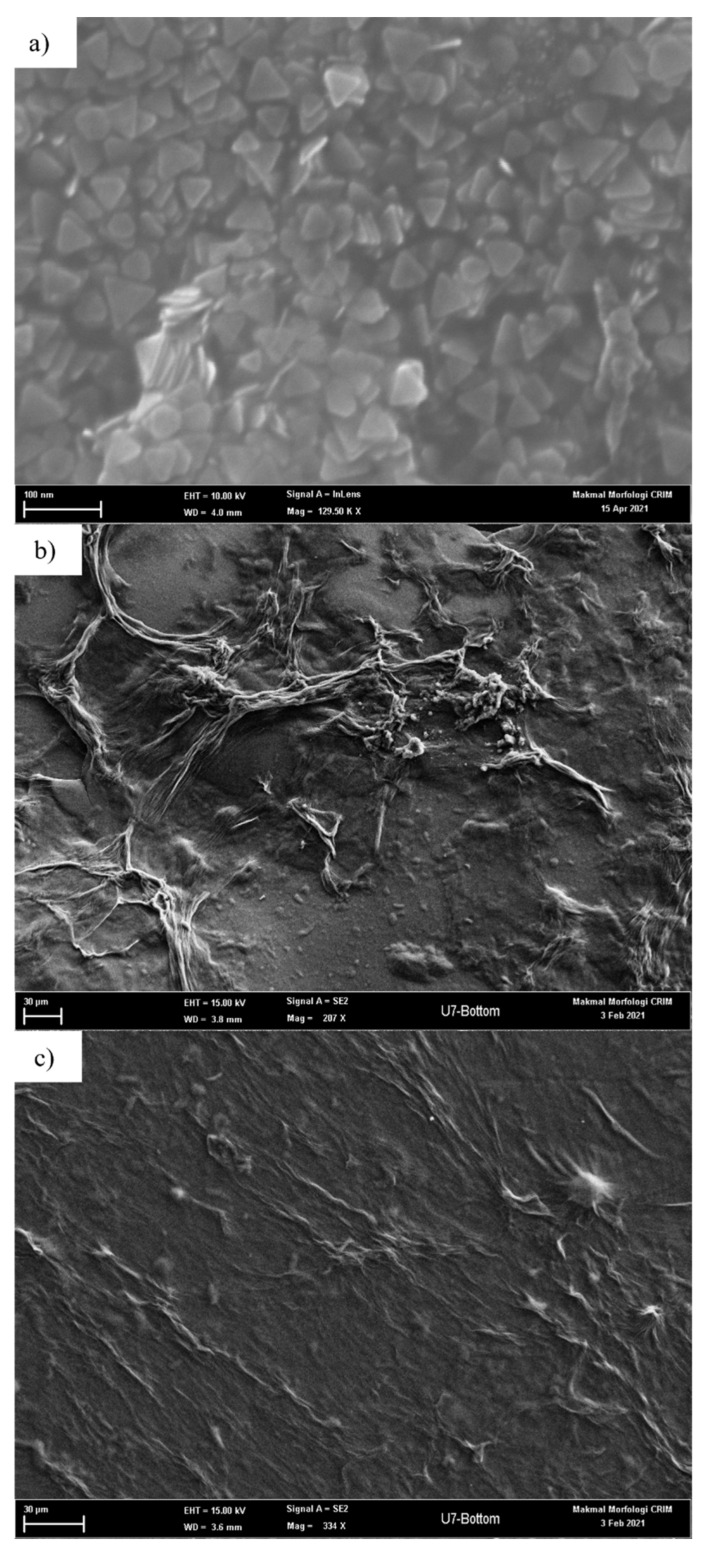
FESEM surface morphologies of the (**a**) AgNP (**b**) κ-carrageenan and (**c**) succinyl-κ-carrageenan films.

In LSPR, AgNP was used to generate intense LSPR responses. In this study, AgNP was combined with κ-carrageenan and succinyl-κ-carrageenan, respectively, to increase the performance of LSPR. To further understand how the sensing material interacts with NH_4_^+^, FESEM was used to carry out the morphological study. The surface morphologies of bare AgNP, κ-carrageenan and succinyl-κ-carrageenan substrate are shown in Figure 3. Figure 3a shows the FESEM image of triangular nanoparticle were successfully coated on the surface of glass substrate proving the fruitful synthesis of AgNP in the form of triangular shape using procedure reported in [9,10]. Meanwhile, Figure 3b shows the surface morphology of the κ-carrageenan film with uneven and featureless topology compared to succinyl-κ-carrageenan. The surface morphology of succinyl-κ-carrageenan (Figure 3c) was even and smooth.

**Figure 4:**
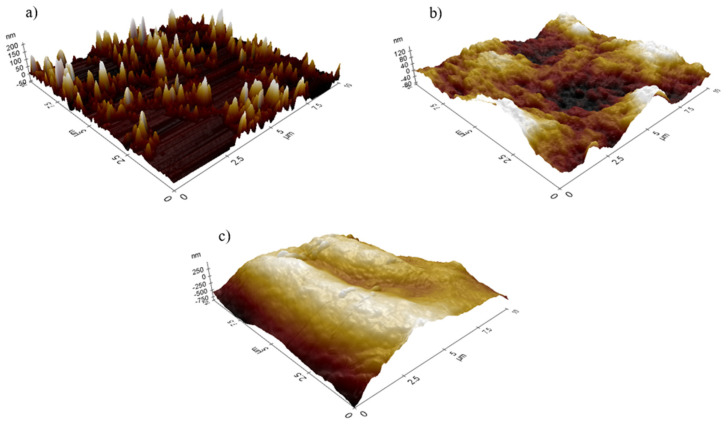
Surface roughness of the (**a**) bare AgNP, (**b**) AgNP-κ-carrageenan, and (**c**) AgNP-succinyl-κ-carrageenan films.

Figure 4 illustrate the three-dimensional (3D) image of (a) AgNP, (b) AgNP-κ-carrageenan, and (c) AgNP-succinyl-κ-carrageenan on the surface of a salinized glass substrate using AFM. The variation topology can be differentiated by calculating the average roughness (r_a_) of each figure using XE Image Processing software. The average roughness of bare AgNP, AgNP-κ-carrageenan and AgNP-succinyl-κ-carrageenan (Table 1). Based on r_a_ value, the surface roughness increased after AgNP was incorporated with κ–carrageenan and succinyl-κ–carrageenan. Furthermore, it was observed that the average surface roughness of AgNP-succinyl-κ-carrageenan in Figure 4c was up to seven times rougher as compared to AgNP-κ-carrageenan in Figure 4b. This could be attributed to the extra oxygen atom in succinyl-κ–carrageenan compared to the structure of κ–carrageenan as confirmed by the FTIR in research conducted by [7]. This large difference in r_a_ was also contributed to by the high degree of substitution during synthesis of succinyl-κ-carrageenan whereby more than one hydroxyl group in pristine κ-carrageenan was substituted with the higher oxygen atom succinyl group [7]. This research pattern was reported previously, whereby adding oxygen-containing compounds increases the surface roughness up to 15 times compared to precursor compound [4,15,16,17]. The result from the average surface roughness from the use of succinyl-κ–carrageenan as sensing material is essential for further study of sensitivity improvement, whereby extra site of C=O from carbonyl group in succinyl moieties provide more interaction with NH_4_^+^, hence leading to higher sensor sensitivity [3,17].

### 3.2. Detection of NH_4_^+^ Using LSPR Technique

**Figure 5:**
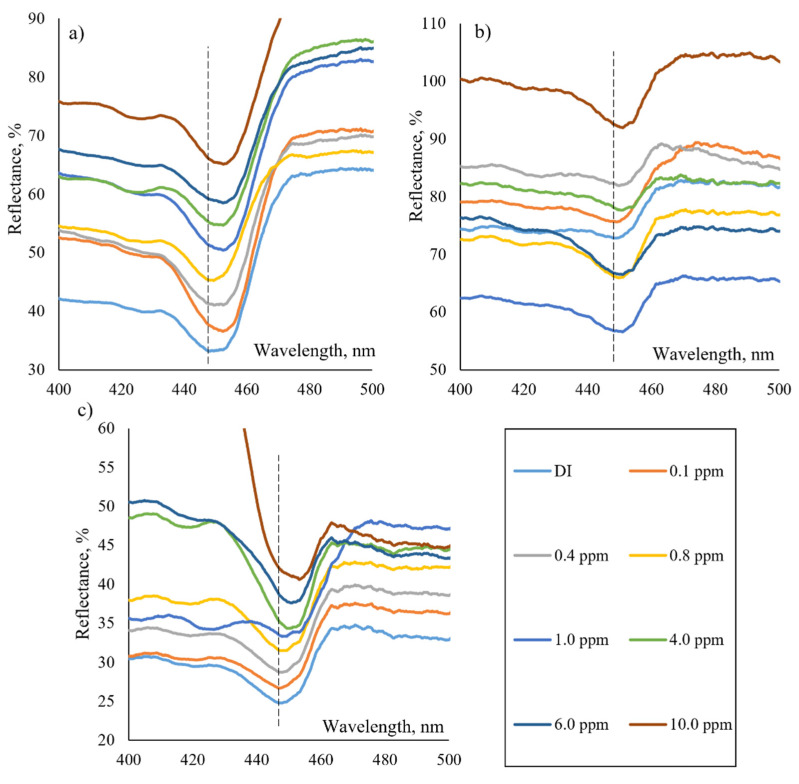
LSPR spectra for detection of NH_4_^+^ at different concentration using (**a**) AgNP, (**b**) AgNP-κ–carrageenan, and (**c**) AgNP-succinyl-κ–carrageenan composites.

**Figure 6:**
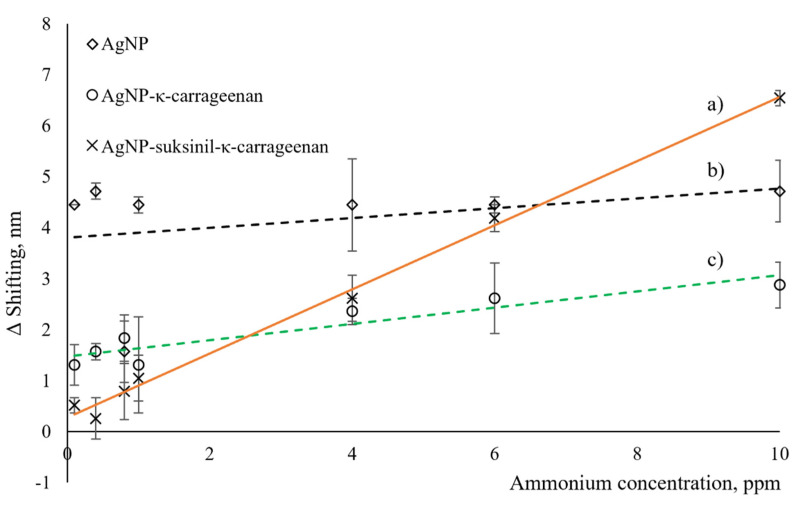
A linear relationship of (**a**) AgNP-succinyl-κ-carrageenan composite (**b**) bare AgNP and (**c**) AgNP-κ–carrageenan composite for NH_4_^+^ sensing in the range of 0.1–10 ppm concentration.

AgNP-κ–carrageenan and AgNP-succinyl-κ–carrageenan composites were used as sensing materials to develop the LSPR sensor to detect NH_4_^+^. The performance of these two materials was compared with bare AgNP based on peak wavelength shift. The present study used deionized water (DI) as the baseline for bare AgNP, AgNP-κ–carrageenan, and AgNP-succinyl-κ–carrageenan composites. Figure 5a reveals the spectrum of bare AgNP, which shows the narrow peak at different concentrations of NH_4_^+^. However, the bare AgNP does not show any distinct peak shifting ranging from 0.1–10.0 ppm. Meanwhile, Figure 5b represents the spectrum of AgNP-κ–carrageenan composite with distinct peak shifting when exposed to the different NH_4_^+^ concentrations. The reflectance peak is red-shifted to 449.42, 449.68, 449.95, 449.42, 450.47, 450.73, and 450.99 from the baseline (448.11 nm) for 0.1, 0.4, 0.8, 1.0, 4.0, 6.0, and 10.0 ppm, respectively. Figure 5c represents the LSPR spectrum of AgNP-succinyl-κ–carrageenan composite when tested with different concentrations of NH_4_^+^. The peak spectra of the reflectance are red-shifted to 446.8, 447.32, 447.85, 447.59, 447.85, 449.42, 450.99, and 453.35 nm from the baseline (446.8 nm) corresponding to 0.1, 0.4, 0.8, 1.0, 4.0, 6.0, and 10.0 ppm, respectively. A relationship study was conducted to validate the LSPR sensor’s performance for bare AgNP, AgNP-κ–carrageenan and AgNP-succinyl-κ–carrageenan and the sensitivity of materials were studied as depicted in Figure 6.

The linear relationship study was carried out between bare AgNP, AgNP-κ–carrageenan, and AgNP-succinyl-κ–carrageenan composites with NH_4_^+^ ion concentration, ranging from 0.1–10.0 ppm. Figure 6 demonstrates the wavelength shift (Δλ) for bare AgNP, AgNP-κ–carrageenan, and AgNP-succinyl-κ–carrageenan composites for thrice measurements with different concentrations of NH_4_^+^ ion. As a baseline, DI water was used to analyse the correlation coefficient (R^2^), and the sensor’s sensitivity was determined from the slope of the graph [3,18]. Figure 6a observed that AgNP-succinyl-κ–carrageenan composite shows excellent linearity with R^2^ = 0.9947 when using sensing material to detect various concentrations of NH_4_^+^ ion. In addition, the sensitivity of the AgNP-succinyl-κ–carrageenan LSPR sensor was calculated to be 0.626 nm ppm^−1^. Temporarily, Figure 6b,c show the graph of bare AgNP and AgNP-κ–carrageenan composite with the linearity of R^2^ = 0.0999 and R^2^ = 0.8662, and the calculated sensitivity of 0.0692 and 0.1061 nm ppm^−1^, respectively. Enhanced sensitivity was detected for AgNP-succinyl-κ–carrageenan composite compared to AgNP-κ–carrageenan composite and bare AgNP due to the higher density of electronegative atom in the succinyl-κ–carrageenan functional group compared to κ–carrageenan, as confirmed by AFM in Figure 4 and FTIR reported in [7]. These extra electronegative oxygen atoms provided more binding sites for NH_4_^+^ ions; hence, higher sensitivity was achieved for AgNP-succinyl-κ–carrageenan compared to AgNP-κ–carrageenan. This phenomenon pattern was also described previously, where the substitution of the κ-carrageenan functional group with a more electronegative group increases the performance of the polysaccharides [6,7,8].

Furthermore, it was observed that AgNP-succinyl-κ–carrageenan shows lower limit of detection (LOD) and limits of quantification (LOQ) compared with AgNP-κ–carrageenan, as shown in Table 2. This is due to the stronger interaction between AgNP-succinyl-κ–carrageenan with NH_4_^+^ compared to AgNP-κ–carrageenan [7]. Based on the LSPR sensor performance results, it is proven that AgNP-succinyl-κ–carrageenan composite shows better performance toward NH_4_^+^ ion detection than bare AgNP and AgNP-κ–carrageenan composite.

When compared to the standard approach for NH_4_^+^ detection using Nessler and indophenol reagent, AgNP-succinyl-κ–carrageenan LSPR has a broader detection range, detecting up to 10 ppm. Meanwhile, the Nessler and indophenol reagent standard methods can only detect NH_4_^+^ up to 5 and 0.160 ppm, respectively [19]. In addition, the Nessler and indophenol reagent approaches are complex. They contain the hazardous mercury salt reagent and phenol, which is toxic. The present work can detect NH_4_^+^ at a lower concentration range compared to other techniques such as electrochemical and colorimetric techniques. The proposed LSPR technique also comply with the detection range of NH_4_^+^ stated by the World Health Organization (WHO) [20,21].

**Figure 7:**
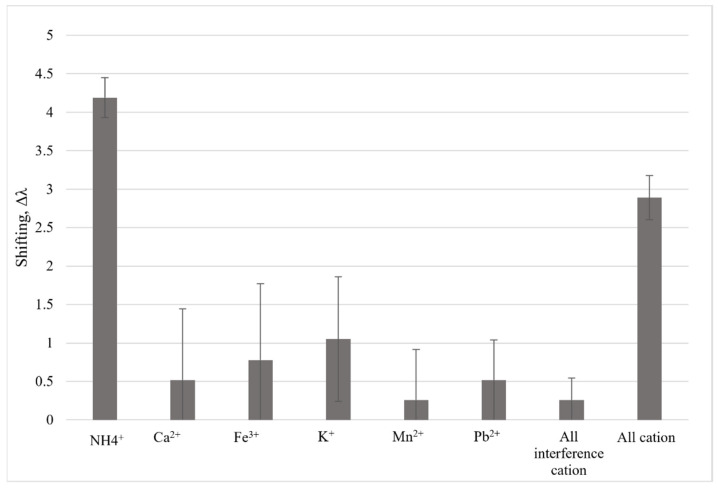
Selectivity test for AgNP-succinyl-κ–carrageenan LSPR.

The selectivity test was performed to determine the sensing materials’ affinity for the target analyte. To investigate the possibility of interference from other cations, the glass substrate coated with AgNP-succinyl-κ-carrageenan was exposed to solutions containing other contaminant cations such as calcium (Ca^2+^), iron (Fe^3+^), potassium (K^+^), manganese (Mn^2+^), and lead (Pb^2+^) at 6 ppm. Figure 7 indicates that the sensor probe has high selectivity for NH_4_^+^ ions compared to other cations, as shown by the major difference in wavelength shift for NH_4_^+^. This might be due to the formation of extra hydrogen bonds between hydrogen in NH_4_^+^ and oxygen atom in the succinyl-κ-carrageenan structure. This hydrogen bond does not occur in other cation interference. This phenomenon is also supported by a few research whereby the hydrogen atom in NH_4_^+^ can interact with electronegative oxygen atom via hydrogen bond [7,22,23].

### 3.3. Interaction Mechanism Study

**Figure 8:**
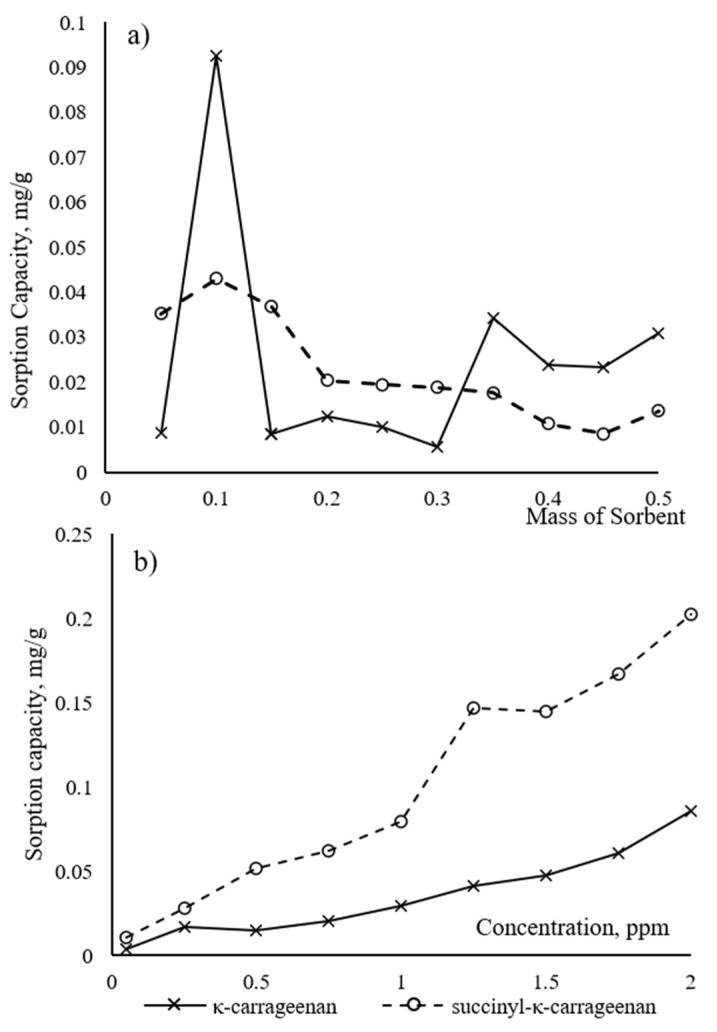
Effect of (**a**) adsorbent dosage on NH_4_^+^ (**b**) initial NH_4_^+^ concentration for NH_4_^+^ adsorption on κ-carrageenan and succinyl-κ-carrageenan.

The isotherm and kinetic model were used to study the interaction mechanism. The effect of pristine κ–carrageenan and succinyl-κ-carrageenan dosage on the adsorption capacity of NH_4_^+^ was shown in Figure 8a. The highest capacity adsorption of NH_4_^+^ was observed at 0.1 g, where succinyl-κ-carrageenan was 0.10 mg/g while κ-carrageenan was 0.04 mg/g. These phenomena showed that succinyl-κ-carrageenan could adsorb a higher amount of NH_4_^+^ compared to pristine κ-carrageenan. This proves that substituting succinyl group in κ-carrageenan provide more active sites for the interaction with NH_4_^+^. However, the adsorption capacity was gradually decreasing with the increase of adsorbent mass. This is due to the accumulation or overlapping of the active site onto the sorbent for both compounds. A similar result was also reported during the adsorption of arsenic ion on Fe_3_O_4_/sugarcane bagasse activated carbon and aniline on graphene oxide, in which the increase of arsenic ion concentration leads to the reduction of adsorption capacity [12,24,25].

Meanwhile, the effect of initial NH_4_^+^ concentration is depicted in Figure 8b. The adsorption capacity increased with the increase of NH_4_^+^ concentration for both compounds. The effective collision increased with the increase of NH_4_^+^ concentration. A similar trend was also observed during the adsorption of Cd^2+^ and Pb^2+^ ions on chitosan and carboxymethyl cellulose. The heavy metal ion adsorption was first increased in response to the regular increases in metal ion concentrations [24,26]. At the concentration of 2 ppm NH_4_^+^, the adsorption capacity of NH_4_^+^ onto succinyl-κ-carrageenan is twice higher than κ-carrageenan. This is due to higher electronegative density in succinyl-κ-carrageenan compared to κ-carrageenan, that act as an extra available site for sorption of NH_4_^+^, hence, increased the performance of NH_4_^+^ sorption. Enhancement of derivative compound performance also has been reported using modified chitosan. After modification, the metal ion adsorption capacity of modified chitosan was increased four times compared to unmodified chitosan [27].

**Figure 9:**
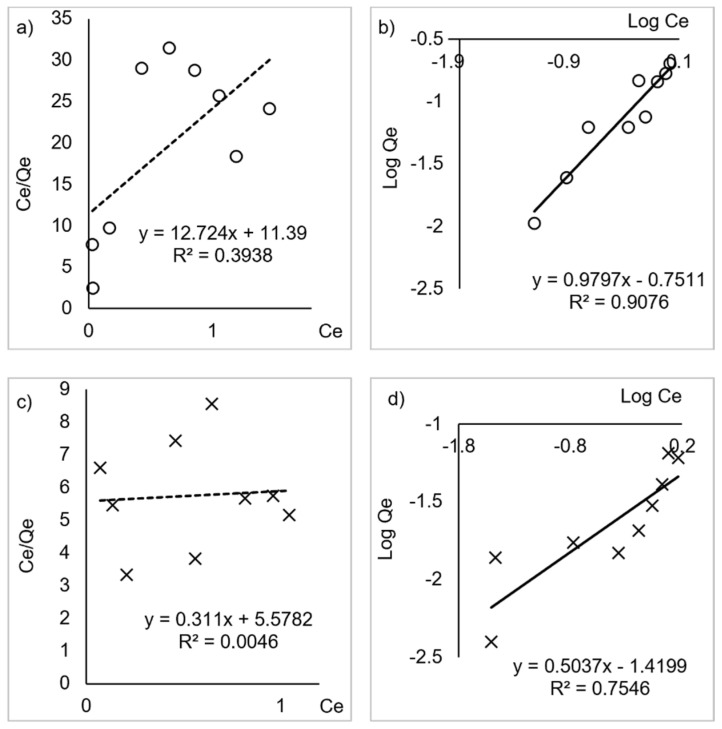
The linearized (**a**) Langmuir isotherm of κ-carrageenan, (**b**) Freundlich isotherm of κ-carrageenan, (**c**) Langmuir isotherm of succinyl-κ-carrageenan, (**d**) Freundlich isotherm of succinyl-κ-carrageenan.

Adsorption isotherm is an essential study describing how NH_4_^+^ interact with the surface of κ-carrageenan derivatives. Therefore, theoretical equations or empirical equations to correlate equilibrium data are necessary for adsorption understanding and adsorption degree estimation. Langmuir and Freundlich isotherm model equations can be used to interpret the obtained adsorption data. A linear plot of C_e_/q_e_ versus C_e_ for Langmuir model and a linear plot of Log Q_e_ versus Log C_e_ for Freundlich model NH_4_^+^ adsorption on κ-carrageenan and succinyl-κ-carrageenan was shown in Figure 9a–d. The isotherm parameter was listed in Table 3. The linear regression of Freundlich isotherm presented the best fit to experimental data for both compounds as the correlation coefficient obtained is higher than Langmuir isotherm. This suggested that NH_4_^+^ were adsorbed onto the κ–carrageenan and succinyl-κ–carrageenan at multilayer adsorption with heterogenous energy distribution surface [11]. The value of 1/*n* was less than 1 for both compounds, implying that chemisorption is favourable for the NH_4_^+^ range being studied [11]. From isotherm and adsorption study, it can be construed that succinyl-κ–carrageenan has higher NH_4_^+^ adsorption capability than κ–carrageenan. It is due to the higher electronegative atom density of succinyl-κ–carrageenan compared to κ–carrageenan as supported by the AFM result (Figure 4).

**Figure 10:**
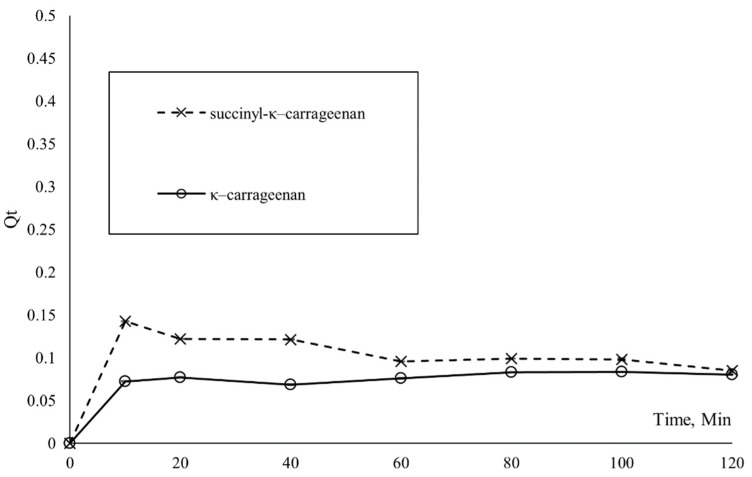
Adsorption of NH4+ onto κ–carrageenan and succinyl-κ–carrageenan as a function of time.

Meanwhile, Figure 10 shows the adsorption of NH_4_^+^ onto κ–carrageenan and succinyl-κ–carrageenan as a function of time. The adsorption capacity of NH_4_^+^ onto κ–carrageenan and succinyl-κ–carrageenan has reached its maximum at 10 and 60 min, respectively, and no significant adsorption changed afterwards until 120 min. The constant adsorption capacity started at the 10th minute for κ–carrageenan and 60th minute for succinyl-κ–carrageenan is probably due to the total occupancy of active sites on adsorbent of both compounds. At this stage, the rate of adsorption and desorption of NH_4_^+^ from the surface of κ–carrageenan and succinyl-κ–carrageenan are equal [24]. A similar trend was also observed in the adsorption of NH_4_^+^ using a hydrogel, whereby the adsorption reached equilibrium in 10 min. Meanwhile, Cheng et al. 2017 reported that adsorption of NH_4_^+^ onto Na-rich birnessite has reached equilibrium within the first few minutes [28]. The pseudo-first-order and pseudo-second-order kinetic models were utilized to investigate the kinetic mechanism of NH_4_^+^ adsorption onto succinyl-κ–carrageenan and κ–carrageenan.

**Figure 11:**
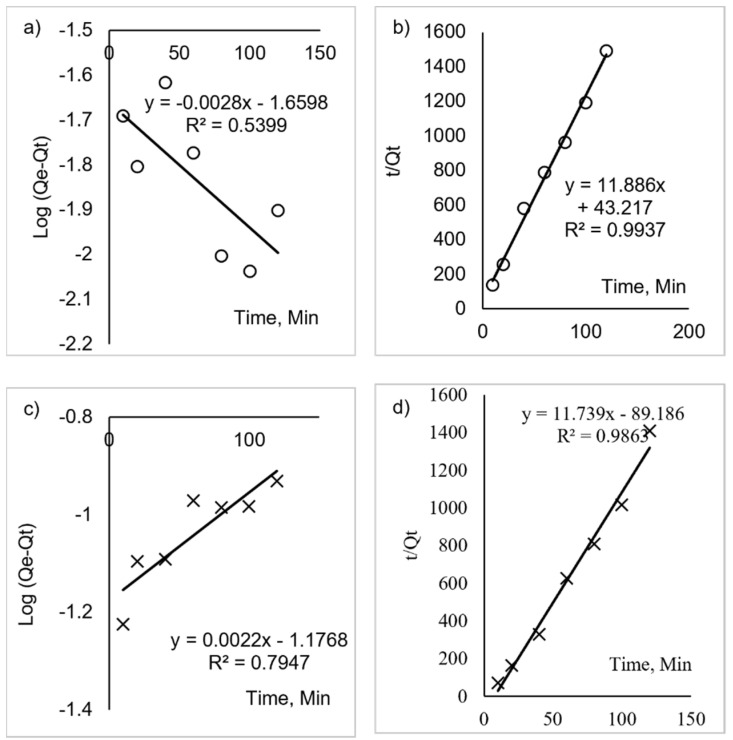
The linearized (**a**) kinetic pseudo-first-order of κ–carrageenan, (**b**) kinetic pseudo-second-order of κ–carrageenan, (**c**) kinetic pseudo-first-order of succinyl-κ–carrageenan, and (**d**) pseudo-second-order of succinyl-κ–carrageenan.

Figure 11a–d shows the linearised pseudo-first-order and pseudo-second-order for κ–carrageenan and succinyl-κ–carrageenan. The calculated q_e_, the rate constants and the correlation coefficient for two kinetic models of κ–carrageenan and succinyl-κ–carrageenan were shown in Table 3. The value of coefficient (R^2^) of pseudo-first-order and pseudo-second-order models for κ–carrageenan were 0.2436 and 0.9822, respectively. The higher R^2^ value for κ–carrageenan indicates the data best fit for the pseudo-second-order model. Meanwhile, succinyl-κ–carrageenan shows higher R^2^ for the pseudo-second-order model than pseudo-first-order with the value of R^2^ 0.9863 and 0.2781, respectively, indicating the data was well fitted with pseudo-second-order. Pseudo-second-order assumed that adsorption of NH_4_^+^ onto κ–carrageenan and succinyl-κ–carrageenan was through chemisorption supporting the result from Freundlich model in isotherm study [29]. It also suggested that the adsorption of NH_4_^+^ onto succinyl-κ–carrageenan and pristine κ–carrageenan obeyed the pseudo-second-order kinetic indicated that the adsorption mechanism depended on the compound dosage and NH_4_^+^ concentration. Pseudo-second-order suggested the initial adsorption of NH_4_^+^ onto succinyl-κ–carrageenan increase with the increase of NH_4_^+^. The lower the concentration of NH_4_^+^ in the solution, the lower the effectiveness of NH_4_^+^ collision with the succinyl-κ–carrageenan [13]. This finding was also observed in other research using biopolymer adsorbent, whereby the adsorption of methylene blue onto carboxymethyl cellulose/k-carrageenan/activated montmorillonite composite beads and agar/κ-carrageenan composite hydrogel follows pseudo-second-order of reaction [14,30].

## 4. Conclusions

Succinyl-κ–carrageenan was successfully investigated for the enhancement of the NH_4_^+^ LSPR sensor. Succinyl-κ–carrageenan was synthesised, and the performance of succinyl-κ–carrageenan toward NH_4_^+^adsorption was compared with pristine κ–carrageenan. Based on the morphology analysis by FESEM, succinyl-κ-carrageenan shows an even and featureless topology; meanwhile, κ-carrageenan displays an uneven surface with a wrinkle-like surface. Succinyl-κ–carrageenan was composited with AgNP on the salinized glass substrate for enhancement of LSPR sensor toward NH_4_^+^. AFM analysis demonstrates that the average surface roughness of AgNP-succinyl-κ-carrageenan is far higher than the AgNP-κ-carrageenan with the value of 198.535 nm and 27.265 nm, respectively. The performance of the fabricated LSPR sensor toward NH_4_^+^ using AgNP-succinyl-κ-carrageenan as active material is better compared to AgNP-κ-carrageenan. AgNP-succinyl-κ-carrageenan LSPR shows higher sensitivity toward NH_4_^+^ compared to AgNP-κ-carrageenan LSPR. In addition, it offers a lower detection and quantification limits with the value of 0.5964 ppm and 2.7192 ppm, respectively.

Furthermore, the fabricated LSPR sensor using AgNP-succinyl-κ-carrageenan as sensing material has a better and broader range of detection up to 10 ppm compared to the standard method. Selectivity test showed AgNP-κ-carrageenan LSPR is highly selective toward NH_4_^+^. The interaction mechanism study shows that the adsorption of NH_4_^+^ onto succinyl-κ–carrageenan and pristine κ–carrageenan is well fitted Freundlich isotherm model and pseudo-second reaction suggesting the adsorption mechanism is through multilayer and chemical process. It is expected that the fabricated NH_4_^+^ LSPR sensor based on biopolymer is highly potential to be used in future research, such as fiber optic LSPR sensor with the advantage of miniature, low cost, in situ and simple sensor technique.

## Figures and Tables

**Table 1:** Average surface roughness of bare AgNP, AgNP-κ-carrageenan, and AgNP-succinyl-κ-carrageenan.

Compound	Average Surface Roughness, r_a_ (nm)
AgNP	20.777
AgNP-κ–carrageenan	27.265
AgNP-succinyl-κ–carrageenan	198.535

**Table 2:** The performance and correlation coefficient R^2^ of AgNP, composite AgNP-κ–carrageenan and composite AgNP-succinyl-κ–carrageenan for NH4+ detection.

Compound	Sensitivity (nm ppm^−1^)	Regression (R^2^)	LOD (ppm)	LOQ (ppm)	Range of Detection (ppm)	Reference
AgNP	0.0962	0.0999	20.7618	62.9146	-	This work
AgNP-κ–carrageenan	0.1601	0.8662	2.7192	8.2400	2.7–6.0	This work
AgNP-succinyl-κ-carrageenan	0.6289	0.9947	0.5964	2.7192	0.6–10.0	This work
Standard Method Indophenol Reagent	-	-	0.160	-	0.35–1.8	[19]
Standard Method Nessler Reagent	-	-	0.6	-	0.85–5.0	[19]
PANi, CPANI, Ag (electrochemical)	-	-	-	-	3.6–3550	[20]
μPAD (colorimetric pH indicator)	-	0.999	0.47	-	2.0–10.0	[21]

**Table 3:** Summary of adsorption isotherm and kinetic variable.

Isotherm and Kinetic Model	Variable	κ-Carrageenan	Succinyl-κ-carrageenan
Langmuir	Q_max_ (mg/g)	0.053	3.215
	K_L_ (L/mg)	2.1679	0.0558
	R_L_	0.8218	0.9471
	R^2^	0.6138	0.0046
Freundlich	K_F_ (g mg^−1^ min^−1^)	0.0380	0.1774
	1/n	0.5037	0.9797
	R^2^	0.7546	0.9076
Pseudo-first-order	q_e_ (mg/g)	0.9911	1.0051
	k_1_ (min^−1^)	3.8096	2.7102
	R^2^	0.2436	0.7947
Pseudo-second-order	q_e_ (mg/g)	0.0193	0.0112
	k_2_ (g min^−1^)	229.0380	677.8876
	R^2^	0.9822	0.9863

## Data Availability

Not applicable.
